# Cytotoxic lesion of the corpus callosum (CLOCC) associated with typhoid fever: A case report

**DOI:** 10.1016/j.radcr.2025.07.034

**Published:** 2025-08-19

**Authors:** Mehdi EL Azouzi, Amine Bentahar, Abdelghani EL Fikri

**Affiliations:** a Radiology Department, Military Hospital Mohammed V, Hay Ryad, 10100, Rabat, Morocco; b Radiology Department, Military Hospital Moulay EL Hassan,Mohammed VI Avenue, 81000, Guelmim, Morocco

**Keywords:** Cytotoxic lesions of the corpus callosum, Magnetic resonance imaging, Typhoid fever

## Abstract

Cytotoxic lesions of the corpus callosum (CLOCCs) are rare but significant abnormalities characterized by transient lesions in the splenium of the corpus callosum, often reversible after the treatment of the underlying condition. These lesions, first described in 1990, are associated with various aetiologies, including infections, metabolic disorders, and trauma. In this context, we report the case of a 44-year-old man with typhoid fever and neurological manifestations, in whom cerebral imaging revealed a cytotoxic lesion of the corpus callosum, thus highlighting this rare pathology.

## Introduction

Cytotoxic lesions of the corpus callosum (CLOCCs) are rare and transient abnormalities [1], typically located in the splenium of the corpus callosum, and can be identified on magnetic resonance imaging (MRI) by diffusion restriction [2]. These lesions can occur for various reasons, such as infections, metabolic disorders, seizures, or trauma. They are generally reversible once the underlying cause is treated [3].

## Case report

A 44-year-old male patient, without any significant prior medical history. was admitted to the emergency department with a 5-day persistent fever of up to 40°C, partially reduced by the administration of paracetamol. The fever was accompanied by abdominal pain, headache, and multiple episodes of watery, fetid stools without blood, which occurred on several occasions. The patient had recently travelled to a sub-Saharan African country and had been back for a fortnight. Before his departure, he had not visited a travel clinic and had not received any specific vaccination recommendations.

Physical examination revealed a patient in poor general condition, febrile at 39.5°C, tachycardic (160 beats per minute), with a blood pressure showing a large pressure difference (112/55 mmHg). Biological tests revealed a C-reactive protein (CRP) level of 230 mg/l, an elevated sedimentation rate of 50 mm/h, alkaline phosphatases of 400 IU/l, an aspartate aminotransferase level of 90 U/L and an alanine aminotransferase level of 70 U/L. The blood count showed a hyperleukocytosis of 14,000/mm3, with 73% neutrophils. Two successive blood cultures revealed the presence of Salmonella typhi, confirming the diagnosis of typhoid fever. The patient was admitted to the Infectious Diseases Department and began appropriate antibiotic therapy.

During the course of the disease, on the 5th day, the patient presented with a state of torpor, interspersed with dreamlike delirium, with nocturnal insomnia, but without meningeal signs or focal neurological signs.

**Fig. 1:**
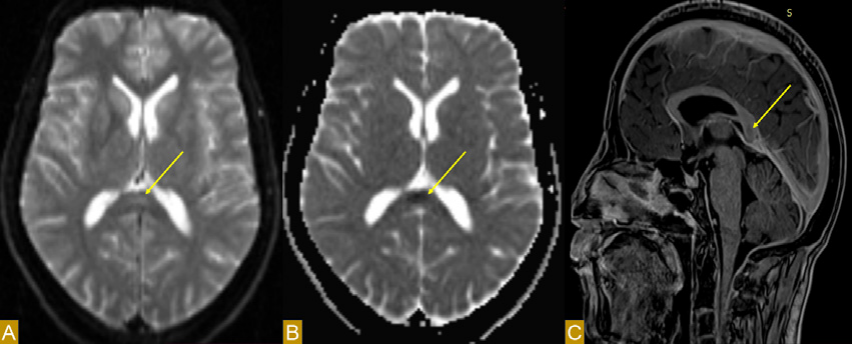
Brain MRI showing a well-defined, midline focal lesion in the splenium of the corpus callosum (arrow), characterized by hyperintensity on the diffusion sequence (A) and a drop in signal on the apparent diffusion coefficient map (B), with no enhancement after gadolinium injection (C).

Magnetic resonance imaging (MRI) revealed a focal lesion of the splenium of the corpus callosum, of median topography, well circumscribed, measuring 20 mm in long axis, without any notable mass syndrome. This lesion appeared hypersignal on FLAIR/T2 weighted images and slightly hyposignal on T1-weighted images. Diffusion restriction (fall in ADC - Apparent Diffusion Coefficient) was present, suggesting cytotoxic oedema. There was no evidence of haemorrhage or calcification in this lesion. After injection of gadolinium, there was no enhancement. The rest of the brain scan was normal (Fig. 1).

Because of the aspecific nature of the lesion, a close iterative check was carried out 1 week later, using the same imaging modality. This revealed significant morphological and signal changes in the callosal lesion.

**Fig. 2:**
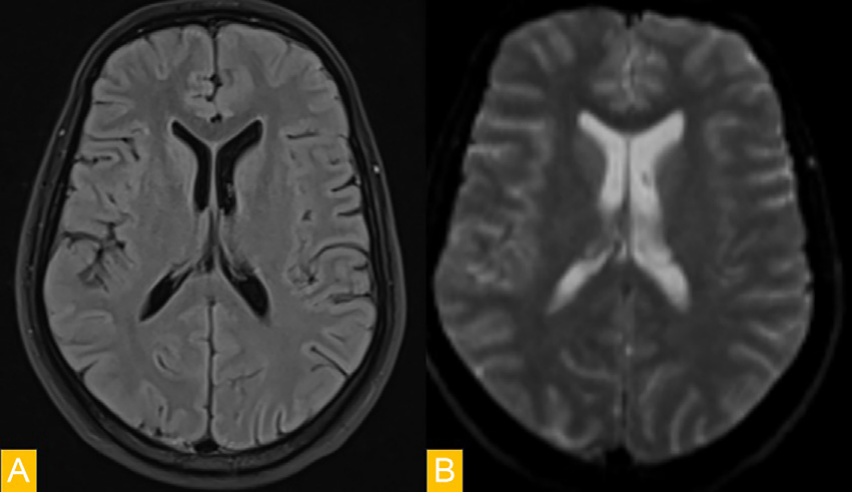
Follow-up MRI demonstrating complete resolution of the splenial lesion. No abnormal signal is seen on FLAIR (A) or diffusion-weighted imaging (B), confirming the transient nature of the lesion.

The patient's clinical and mental state subsequently improved, and a further follow-up MRI showed a favourable course with no residual abnormalities (Fig. 2). Given the transient nature of this lesion, the diagnosis of cytotoxic lesion of the corpus callosum was retained. In this case, the phenomenon appears to be secondary to infection (typhoid fever).

## Discussion

Cytotoxic lesions of the corpus callosum (CLOCCs) are a rare clinical and radiological syndrome (Table 1) [3], characterised by transient lesions in the splenium of the corpus callosum, with diffusion restriction on MRI [2]. These lesions were first described in Japan in 1990, but the terminology has evolved over the years [2]. Prior to the adoption of the term CLOCC, several other terms were used, including “transient lesions of the splenium of the corpus callosum”, “mild encephalitis with reversible lesions of the splenium of the corpus callosum (MERS)”, and “syndrome of reversible splenial lesions (RESLES)”[1]. The term CLOCC better reflects the underlying pathophysiology of these lesions, emphasising that they may not be confined to the splenium and are often reversible after resolution of the underlying pathology [3].

**Table 1:** Clinical and radiological criteria for cytotoxic lesions of the corpus callosum [3].

Clinical and radiological criteria for CLOCCs
1. The onset of neuropsychiatric symptoms such as abnormal speech and/or behavior, as well as altered consciousness or seizures, within a week of the onset of fever.
2. Complete recovery without sequelae, mainly within 10 days after the onset of neuropsychiatric symptoms.
3. High signal intensity lesion in the splenium of the CC (during the acute stage). Signal changes on T1 and T2 are light.
4. The lesion may involve the entire CC and cerebral white matter symmetrically.
5. The lesion disappears within a week, with no residual signal changes or atrophy.

CLOCCs can be observed in a variety of clinical settings, including viral infections, metabolic disorders, seizures, drugs, malignancies, and trauma (Table 2) [1]. Salmonella typhi infection, as in our clinical case, is 1 of the identified causes of CLOCCs. Other infections, such as influenza, rotavirus and herpes, have also been associated with these lesions.

**Table 2:** Causes of cytotoxic lesions of the corpus callosum [1].

Causes of CC cytotoxic lesion	Cause
Entity	
Drugs	Carbamazepine, Cyclosporine, Intravenous immunoglobulin, Lamotrigine, Metronidazole, Amitriptyline, Clozapine, Phenytoin, Corticosteroids
Malignant neoplasms	Acute lymphocytic leukemia, Esophageal cancer, Leptomeningeal glioblastomatosis, Spinal meningeal melanocytoma
Infections	Adenovirus, Aseptic meningitis, Encephalitis, Epstein-Barr virus, Escherichia coli, Herpes, Influenza virus A (H1N1), Legionella, Malaria, Measles, Mycoplasma, Mumps, Rotavirus, Salmonella, Staphylococcus, Streptococcus, Varicella-zoster virus
Subarachnoid hemorrhage	Aneurysm, Arteriovenous malformation
Metabolic disorders	Acute kidney failure, Alcoholism, Extrapontine myelinolysis, Central pontine myelinolysis, Hepatic encephalopathy, Hyperammonemia, Hypernatremia, Hypoglycemia, Hyponatremia, Malnutrition, Wernicke’s encephalopathy, Wilson’s disease, Marchiafava-Bignami disease
Trauma	Secondary trauma from different causes
Other entities	Acute altitude sickness, Antiglutamate receptor antibodies, Antivoltage gated potassium channel antibodies, Eclampsia, Hemolytic uremic syndrome, Vaccination, Kawasaki disease, Posterior reversible encephalopathy syndrome, Postpartum cerebral angiopathy, Epileptic status

CLOCCs appear to result from a cascade of cytokines and cellular stimuli (cytokinopathy), which are triggered by infection, inflammation or trauma. This cascade begins with the release of inflammatory cytokines (eg, interleukin-1 (IL-1) and interleukin-6 (IL-6)) by macrophages, leading to a series of changes, including the activation of T lymphocytes, the breakdown of the blood-brain barrier and the production of tumour necrosis factor alpha (TNF-α). This activation leads to a massive influx of glutamate, which causes cytotoxic oedema in neurons, astrocytes and oligodendrocytes [1].

The corpus callosum, especially its splenium, is particularly vulnerable to this phenomenon because of the high density of receptors for cytokines, glutamate and excitatory amino acids. Thus, cytotoxic oedema tends to develop in this region of the brain when an inflammatory cascade is triggered [4].

MRI is the ideal diagnostic tool for observing CLOCCs [5]. On MRI, CLOCCs show 3 main patterns depending on their shape and extent [1,5]:

1. Small, well-defined oval lesions: This is the most common pattern and is typical of patients with epileptic seizures or metabolic disorders.
2. More extensive and irregular lesions: These lesions often extend across the splenium and into adjacent hemispheres.
3. Posteriorly centred lesions extending into the anterior part of the corpus callosum.

In general, lesions are hyperintense on T2 and FLAIR sequences and show diffusion restriction (reduced ADC values) [5]. This phenomenon is an indicator of cytotoxic oedema, which is reversible in most cases, once the underlying cause has been controlled [6].

The prognosis of CLOCCs is generally good [7]. In the majority of cases, the lesions are reversible and disappear once the underlying disease has been resolved [8]. Treatment of CLOCCs is mainly based on management of the underlying cause, such as bacterial infection, metabolic disorders or seizures. In our case, treatment of typhoid fever with antibiotics resulted in complete resolution of the lesion, and a follow-up MRI showed no sequelae.

The clinical presentation of CLOCCs primarily reflects the underlying pathology rather than the callosal lesion itself [1,9]. Unlike many other lesions of the corpus callosum, CLOCCs are not usually associated with signs or symptoms of hemispheric disconnection, such as pseudoneglect, foreign hand syndrome, left-hand apraxia, agraphia, alexia or visual apraxias. These lesions are often reversible once the underlying disease has been controlled, which is a fundamental aspect of their clinical management. In addition, unlike other aetiologies of corpus callosum lesions, CLOCCs generally do not show enhancement after gadolinium injection, which is a hallmark of splenium involvement as it is in inflammatory or tumour pathologies [6].

CLOCCs must be differentiated from other conditions with similar lesions in the splenium of the corpus callosum [9]. The main pathologies to be considered in the differential diagnosis include demyelination (as in multiple sclerosis), cerebral infarction, posterior reversible encephalopathy syndrome (PRES), head trauma and brain tumours. The use of angiographic sequences and MRI with gadolinium contrast can help to distinguish between these different conditions [10].

## Conclusion

Cytotoxic lesions of the corpus callosum (CLOCCs) represent a clinical and radiological syndrome with various etiologies, leading to cytotoxic edema in the vulnerable regions of the corpus callosum. Over time, the terminology for this condition has evolved, and it is now referred to as CLOCCs. Early detection of CLOCCs helps avoid unnecessary treatments and tests, offering reassurance due to their excellent clinical and radiological prognosis.

## Patient consent

All authors confirm that they have obtained written consent from the patient for publication of this case.

## Author contributions

All authors contributed equally to this work.

## Ethical approval

No ethical approval is required for de-identified single case reports based on our institutional policies.
